# Interactions between Entomopathogenic Fungi and Entomopathogenic Nematodes

**DOI:** 10.3390/microorganisms11010163

**Published:** 2023-01-08

**Authors:** Vladimír Půža, Eustachio Tarasco

**Affiliations:** 1 Biology Centre of the Czech Academy of Sciences, Institute of Entomology, Branišovská 31, 37005 České Budějovice, Czech Republic; 2 Department of Soil, Plant and Food Sciences, University of Bari “Aldo Moro”, Via G. Amendola, 165/a, 70126 Bari, Italy

**Keywords:** entomopathogenic fungi, entomopathogenic nematodes, synergy, biocontrol, effectiveness

## Abstract

Entomopathogenic fungi and entomopathogenic nematodes are globally distributed soil organisms capable of infecting and killing a vast variety of insects. Therefore, these organisms are frequently used as biocontrol agents in insect pest management. Both entomopathogenic fungi and nematodes share the soil environment and thus can infest and compete for the same insect host; however, natural co-infections are rarely found due to the cryptic soil environment. Our current knowledge on their interactions within hosts mainly comes from laboratory experiments. Because of the recent trend of combining biocontrol agents to increase their efficacy, many studies have focused on the co-application of different species of EPF and EPNs against various insect pests with variable outcomes ranging from synergistic effects and additive effects to antagonism. In addition, the effect on the development and reproduction of each pathogen varies from normal reproduction to exclusion, and generally the outcomes of the interactions are dependent on pathogen and host species, pathogen doses, and the timing of infection. The present review aims to summarize the current knowledge on the interactions of entomopathogenic fungi and nematodes within an insect host and to estimate the possible effects of the interactions on natural pathogen populations and on their use in biocontrol.

## 1. Introduction

Entomopathogenic nematodes (EPNs) belonging to the genera *Steinernema* Travassos (Rhabditida: Steinernematidae) and *Heterorhabditis* Poinar (Rhabditida: Heterorhabditidae) are obligate and lethal parasites of insects [1,2]. Their infective juveniles (IJs), non-feeding and usually soil dwelling, hold in their foregut symbiotic bacteria that play an important and essential role in killing susceptible insects. The IJs enter through the insect’s natural openings (mouth, spiracles, and anus) or through the integument in the case of *Heterorhabditis*, and invade the haemocoel through the mid-gut wall and release bacteria; these bacteria provide nutrients and inhibit the growth of other microrgnaisms establishing suitable conditions for nematode reproduction. The associated bacteria multiply rapidly causing septicemia and host’s death after 24–48 h, during which time the nematodes feed on the bacteria and reproduce in the cadaver [3].

Entomopathogenic fungi, mainly Ascomycetes, are regularly found infecting insects in the environment, especially in the soil. The species of the genera *Metarhizium* Sorokin, and *Beauveria* Vuill. are the best known entomopathogenic fungi. These organisms usually attach to the external body of insects by conidia adhering to the host’s cuticle. Under the right temperature and humidity conditions, these spores germinate, grow as hyphae, and colonize the insect’s body. After a few days (4–7), the insect is usually killed, especially by fungal toxins, and new spores are formed in or on the insect (sporulation), ready to be spread in the environment [4].

The combined use of the entomopathogenic fungi (EPF) and nematodes (EPNs) is considered an interesting and effective approach in microbial pest control. The studies conducted so far have focused mainly on the combined use of both groups of biological control agents to increase their insecticidal efficacy under laboratory conditions [5,6,7,8,9,10], while their interactions and the possible performances of EPNs and EPF under natural conditions are largely unknown.

## 2. Interactions between Entomopathogenic Fungi and Nematodes

Both entomopathogenic fungi and nematodes are pathogens/parasites with a broad host range [1,11,12,13,14,15] and they largely share an ecological niche, and often are isolated from the same soil samples. For instance, Tarasco et al. [9] reported co-occurrence of *Steinernema ichnusae* Tarasco, Mracek, Nguyen, and Triggiani (Rhabditida: Steinernematidae) and *Beauveria bassiana* s.s. (*sensu stricto*) (Bals-Criv.) Vuill. (Hypocreales: Cordycipitaceae) in the samples from an oak forest in Sardinia, Italy.

In response to competition, parasites and pathogens exhibit a diverse array of strategies that improve their chances of growth or reproduction over competitors [16]. During their evolution, both entomopathogenic fungi and nematodes have developed multiple strategies for competition with each other, which will be reviewed below.

### 2.1. Nematode Adaptations for Interactions with EPF

One of the common modes of adaptation by nematodes to entomopathogenic fungi involves the avoidance of competition, and this has been demonstrated by Barbercheck and Kaya [17]. In their experiments, a major part of the infective juveniles (IJs) of *Steinernema carpocapsae* (Weiser) (Rhabditida: Steinernematidae) and *Heterorhabditis bacteriophora* Poinar (Rhabditida: Heterorhabditidae) were repelled from the insects infected with *B. bassiana* s.l. (sensu lato). Similarly, in dual infection with the fungus *Cordyceps fumosorosea* (Wize) (Hypocreales: Cordycipitaceae), the invasion rate of *Steinernema feltiae* Bovien (Rhabditida: Steinernematidae) was lower in comparison with the nematode-only application [18]. Nevertheless, in both studies, the avoidance was only partial, as some infective juveniles migrated towards and penetrated the fungus-infected larvae.

Once the nematodes enter the fungus-infested insects, strong competition for resources occurs. Barbercheck and Kaya [19] have shown that the growth of *B. bassiana* s.l. in *Galleria mellonella* L. (Lepidoptera: Pyralidae) was inhibited in dual infection with *S. feltiae*, if the nematodes were applied simultaneously or 12 h after the fungus. The authors suggested that the main cause of inhibition was the bacterial symbiont of *S. feltiae*, *Xenorhabdus bovienii*. Since then, *Photorhabdus* and *Xenorhabdus* bacteria have been shown to produce many compounds with an antifungal activity. For instance, hydroxy–stilbenes (isopropylstilbene) produced by *Photorhabdus luminescens* effectively suppressed fungal human pathogens Aspergillus flavus Link (Eurotiales: Trichocomaceae), Aspergillus fumigates Fresenius (Eurotiales: Trichocomaceae), Botrytis cinerea Pers (Helotiales: Sclerotiniaceae), Candida tropicalis Berkhout (Saccharomycetales: Saccharomycetaceae), and Cryptococcus neoformans (San Felice) Vuill (Tremellales: Tremellaceae) [20]. Gualtieri et al. [21] demonstrated that *Xenorhabdus nematophila* produces antifungal PAX peptides that suppress serious plant and human fungal pathogens. Similarly, the secondary metabolites of *Xenorhabdus budapestensis* and *Xenorhabdus szentirmaii* suppress plant pathogenic fungus *Phytophthora nicotianae* Breda de Haan (Peronosporales: Peronosporacae) [22]. The metabolites from *X. szentirmaii* proved effective against four plant-pathogenic fungi, *Monilinia fructicola*, *Rhizoctonia solani*, *Colletotrichum gloeosporioides,* and *Fusarium oxysporum* [23]. Cimen et al. [24] identified fabclavines as broad spectrum antifungal bioactive compounds responsible for the antifungal activity of *X. szentirmaii*.

The secondary metabolites of *Xenorhabdus* and *Photorhabdus* bacteria were found to be effective during the competition of entomopathogenic nematodes and fungi within insect hosts. For instance, *X. nematophila* inhibited the growth of *B. bassiana* s.l. on agar plates [25]. In another study, *Photorhabdus luminescens* inhibited the growth and conidial production of *Metarhizium anisopliae* (Metch.) Sorokin, *B. bassiana* s.l., *Beauveria brongniartii* (Saccardo) Petch (Hypocreales: Cordycipitaceae), and *C. fumosorosea* [26]. Similarly, Tarasco et al. [9] demonstrated that extracts from *Xenorhabdus bovienii* inhibited the growth of *B. bassiana* s.s.

### 2.2. Fungal Adaptations for the Interactions with EPN

During dual infections with entomopathogenic fungi, numerous studies recorded negative effects on the nematodes as well [7,18,27]. Naturally, entomopathogenic fungi produce many toxic metabolites in order to kill their insect hosts [28,29]; however, some compounds were found to have an antibiotic effect that is believed to protect the fungus against antagonistic microorganisms, or to prevent saprophytic microbes in the host cadaver [30]. Ansari et al. [26] demonstrated that the crude extract of *M. anisopliae* s.l. inhibited the growth of *P. luminescens* and *Xenorhabdus poinarii*. Similarly, fungal extracts from *B. bassiana* s.s. impaired the growth of *X. bovienii* [9].

Recently, Hummadi et al. [31] revealed that entomopathogenic fungus *Metarhizium brunneum* (Petch) (Hypocreales: Clavicipitaceae) produces volatile organic compounds that are highly toxic to the infective juveniles (IJs) of the EPN, *S. carpocapsae*, *S. feltiae*, and *H. bacteriophora*, and these compounds can shape the interaction of these pathogens in the rhizosphere. These findings suggest that the interactions between entomopathogenic fungi and nematodes also occur outside the host.

### 2.3. Outcomes of the Interaction

Both entomopathogenic nematodes and fungi possess numerous adaptations for competition with each other. Barbercheck and Kaya [19] observed that these pathogens rarely co-produce progeny in infected hosts, and one of them usually prevail. The authors also observed that the nematode progeny production decreased with the time between the exposure of the hosts to *B. bassiana* s.l. and nematodes, and the fungus was detrimental to the development of *S. feltiae* and *Heterorhabditis heliothidis* (Khan, Brooks, and Hirschmann) (Rhabditida: Heterorhabditidae) when applied to the insect more than 48 h before nematodes [19]. Barbercheck and Kaya [5] hypothesized that the two day period corresponds to the time that circulating hyphal bodies appear in the fungus-infected host. When nematodes are applied after this period, they are unable to successfully develop in the host, and *B. bassiana* s.l. develops exclusively. Such an exclusion could be attributed to indirect interactions related to competition for the same host resources [32]. Similarly, less virulent strains of *M. anisopliae* s.l. applied 2 days before *H. bacteriophora* decreased nematode reproduction [27].

In simultaneous applications, nematodes usually outcompete the fungus [19]. Ansari et al. [6] observed that in simultaneous application, the combination with *Heterorhabditis megidis* Poinar, Jackson, and Klein (Rhabditida: Heterorhabditidae) and *Steinernema glaseri* Steiner (Rhabditida: Steinernematidae) was totally detrimental for the reproduction of *M. anisopliae* s.l. Interestingly, Shaurub et al. [33] observed the opposite situation, when maximum IJ yields of *Steinernema riobrave* Cabanillas, Poinar, and Raulston (Rhabditida: Steinernematidae) and *H. bacteriophora* were recorded in *Spodoptera littoralis* (Boisduval) (Lepidoptera: Noctuidae) previously exposed to *B. bassiana* s.s. Interestingly, Molina et al. [27] observed that a highly virulent fungal isolate, *M. anisopliae* s.l. totally inhibited the reproduction of *H. bacteriophora* even when applied simultaneously with the nematodes, and reduced nematode reproduction when applied after the nematodes. This observation suggests that the fungus directly interacts with the nematodes via the production of metabolites that are toxic to symbiotic bacteria or nematodes. Toxicity to bacteria is more probable, as the crude extract of *M. anisopliae* s.l. was found to be toxic to bacteria, while it had no toxic effects on *H. megidis* and *S. glaseri* even at the highest concentration [26].

As mentioned above, Barbercheck and Kaya [19] observed that these pathogens rarely co-produce progeny, and this was confirmed by several other studies. For instance, Wu et al. [34] reported that after the joint application of *H. bacteriophora* and *H. megidis* with *B. bassiana* s.s. and *M. anisopliae* s.s., no southern masked chafer white grub, *Cyclocephala lurida* Bland (Coleoptera: Scarabaeidae) showed both fungal sporulation and nematode development. On the other hand, Tarasco et al. [9] observed both *S. ichnusae* and *B. bassiana* s.s. developed in *G. mellonella*. The authors described that both pathogens started the infection process in different parts of the host body and further developed in these defined spaces and competed in the haemocoel to conquer every available space. Therefore, the reproduction of both pathogens within one host is obviously possible, but this phenomenon is likely very rare.

It can be concluded that the interactions between entomopathogenic fungi and nematodes are very competitive and, in general, the nematodes appear to be stronger competitors due to their faster infestation and development inside the host. Nevertheless, in particular pathogen species and strain combinations, the outcome can be different.

### 2.4. Effect of the EPN-EPF Interactions on the Host

As was demonstrated above, the relationship between entomopathogenic fungi and the nematodes is mostly antagonistic, where one or both competitors are negatively affected. Nevertheless, the effect of dual pathogen infection can have an additive or synergistic effect on host mortality and can be used to increase the effectiveness of both pathogens in biological control. Ansari et al. [6] suggested that the mechanism of synergy in the insects infected with the nematodes after the fungus could lie in the fact that fungal infection stresses the host by affecting its food intake and body homeostasis while consequently decreases its mechanisms to overcome nematode infection that are very effective in healthy grubs [35,36]. In addition, the insects infected with the fungus respire more and attract entomopathogenic nematodes that follow gradient of carbon dioxide [8,37].

## 3. Interactions of EPN and EPF in Biocontrol

It is generally accepted that the efficacy of pest control agents can be improved by their combination [38]. According to Jacques and Morris [39] two control agents applied together can act independently of each other in each host, and the impact on the target organism is the sum of the impact of each one resulting in an additive effect. The interaction can be also synergistic or antagonistic, and the combination is thus more or less effective in control than in the case of an additive effect. In synergy, one agent causes the target organism more susceptible to the other; while in antagonistic interaction, the agents are in competition or interfere with each other.

The efficacy of biocontrol agents can be enhanced by their combination with chemical insecticides, as has been shown for the entomopathogenic fungus *M. anisopliae* s.l., which, in combination with a Spinosad product provided synergistic control of exotic wireworms *Agriotes lineatus* L. and *Agriotes obscurus* L. (Coleoptera: Elateridae) [40]. A similar synergistic effect was observed with the same fungus combined with sublethal doses of chemical insecticides in the control of other subterranean pests such as black vine weevil, *Otiorhynchus sulcatus* (F.) (Coleoptera: Curculionidae) [41]. This study documented that the use of a biocontrol agent could significantly reduce the amount of conventional insecticide use and, consequently, decrease the negative effect on the environment.

The efficacy of biocontrol agents can be improved by their combination with another biocontrol agent [42,43] and the same applies for the combination of entomopathogenic fungi with entomopathogenic nematodes. In the last decades, this phenomenon has received increasing attention from many researchers who tested the combination of various EPF species and strains with numerous species and strains of entomopathogenic nematodes against a great variety of insect pests. Most research focused on coleopteran pests from the families Scarabaeidae and Curculionidae (Coleoptera), and fewer studies targeted other coleopteran families or representatives from the orders Lepidoptera and Diptera. Below we summarize the results of this research for each taxonomic group.

### 3.1. Coeloptera Scarabaeidae

In one of the first studies addressing the combination of entomopathogenic fungi and nematodes, Choo et al. [44] evaluated the efficiency of the fungus *B. brongniartii* with two EPNs *S. carpocapsae* and *H. bacteriophora* against the white grubs *Ectinohoplia rufipes* (Motschulsky) and *Exomala orientalis* (Waterhouse) in golf courses in Korea. Interestingly, combining *B. brongniartii* with *S. carpocapsae* resulted in a significant increase in grub mortality over the application of the fungus alone or in comparison with the application of both nematodes *S. carpocapsae* and *H. bacteriophora*. In their study, Choo et al. [44] used only the simultaneous application of both bioagents.

Ansari et al. [6] studied the interaction between the entomopathogenic fungus *M. anisopliae* s.l. and the entomopathogenic nematodes *H. megidis* and *S. glaseri* during co-application against third-instar white grub, *Hoplia philanthus* Füessly, and the authors also assessed different timings for pathogen application. In general, the combination of the fungus with nematodes increased insect mortality and the effect was either additive or synergistic, depending on the dosage and timing of the application, as a stronger synergistic effect was apparent in the insect that had been exposed to *M. anisopliae* s.l. at least 3 or 4 weeks before the nematodes. This highlights the important effect of the timing of application.

The same fungus *M. anisopliae* s.l. was tested in the field conditions for the control of *H. philanthus* in combination with the commercial formulation of entomopathogenic nematode *H. bacteriophora* (Nema-green^®^), and their performance was compared with single pathogen applications and with the chemical insecticide chlorpyrifos [37]. The combination of both pathogens has shown additive or synergistic effects, with the highest pest mortality achieved especially when the nematodes were applied 4 weeks after the application of the fungus. The authors concluded that for the effective control of the pest by the pathogen combination, 50% of the full rate of both components and even less would be sufficient, making the combination of the pathogens a viable approach to improve the control of *H. philanthus* larvae. From an economical point of view, the use of similarly effective organophosphate and carbamate insecticides is cheaper, but has inherent negative non-target effects.

Two entomopathogenic nematodes, *H. bacteriophora* and *Steinernema yirgalemense* Nguyen et al. (Rhabditida: Steinernematidae), and three entomopathogenic fungal isolates of *M. anisopliae* s.l. were applied against the barley chafer grub, *Coptognathus curtipennis* Faimaire, in simultaneous and sequential combinations [45]. The authors observed an additive and synergistic effect of the pathogen combinations with the strongest synergistic effect in the treatment with insects exposed to *M. anisopliae* s.l. weeks before the addition of nematodes. Obviously, the combined use of *M. anisopliae* s.l. with nematodes may offer an integrated approach to increase the efficacy of EPN for *C. curtipennis* control.

Wu et al. [34] combined *H. bacteriophora* and *H. megidis* with entomopathogenic fungi *B. bassiana* s.s. and *M. anisopliae* s.s. in the laboratory and greenhouse experiments against the third instar southern masked chafer white grubs, *Cyclocephala lurida* Bland. In laboratory experiments, additive interactions were found between *H. megidis* and *B. bassiana* s.s., and between *H. bacteriophora* and *B. bassiana* s.s. or *M. anisopliae* s.s. in most combinations, while few treatments showed synergism or antagonism. Surprisingly, there was no effect regarding different timings of pathogen applications. In the greenhouse, an additive or synergistic interaction was found between *H. bacteriophora* and *B. bassiana* s.s. or *M. anisopliae* s.s. in different formulations, again, both in simultaneous applications and when the nematodes were applied 4 weeks after the fungi. Based on the data, the authors stress that the combination of nematodes and fungi may achieve an effect comparable or superior to an imidacloprid insecticide for the curative control of *Cyclocephala lurida,* and recommend the need for more virulent fungal strains to achieve a stronger interaction with the nematodes.

### 3.2. Coeloptera Curculionidae

The combination of the insect-pathogenic fungus *M. brunneum* with entomopathogenic nematodes was also tested for the control of the black vine weevil, *Otiorhynchus sulcatus* Fabricius [8]. The combination of the fungus with *H. bacteriophora*, *S. feltiae*, and *Steinernema kraussei* Steiner (Rhabitida: Steinernematidae) provided increased control of the pest in both the laboratory and greenhouse. The highest control was achieved with the nematodes *S. feltiae* and *H. bacteriophora,* applied 1–2 weeks after the fungus, which showed the highest synergistic effect. Interestingly, for the application of *S. kraussei*, the synergistic effect was present when the nematode was applied simultaneously with the fungus, suggesting the species-specific outcome of the interaction. The greenhouse tests suggested that both *M. brunneum* and the nematodes can be used at significantly lower rates and still provide good control of black vine weevil larvae, while being cost-effective. Therefore, the tested system was deemed to be an economically feasible approach for black vine weevil larval control. In the following study [46], the overwintering black vine weevils in greenhouses were exposed to *M. brunneum* and a cold tolerant strain of *S. kraussei,* which resulted in 100% control of the overwintering pest and the interaction was synergistic in the first trial, but when the trial was repeated, the results were additive. This discrepancy was attributed to the fact that in another trial, *M. brunneum* had already caused a high larval mortality, which did not leave enough room for further significant improvement from the addition of nematodes.

The combined effects of different fungal species of *B. bassiana* s.l., *M. anisopliae* s.l., and *C. fumosorosea* and nematodes *Heterorhabditis indica* Poinar, Karunakar, and David (Rhabditida: Hetrorhabditidae) or *S. carpocapsae* was evaluated for the control of the pecan weevil, *Curculio caryae* (Horn), larvae [7]. No synergy was observed in terms of the observed pest mortality and most of the interactions were either additive or antagonistic, with the latter being more common. Interestingly, the combination of *C. fumosorosea* with the nematodes always resulted in antagonism, while the result of the interaction of two other fungi depended on the nematode species. The interactions between *H. indica* and *B. bassiana* s.l. were antagonistic, whereas the interactions between *H. indica* and *M. anisopliae* s.l. were additive. Interactions between *S. carpocapsae* and *B. bassiana* s.l. or *M. anisopliae* s.l. differed depending on the application rate. Based on these results, the authors concluded that the tested pathogen combinations were not likely to improve the suppression of *C. caryae* larvae in comparison with the single application of the pathogen with greatest virulence. Furthermore, the results highlighted the effect of the pathogen species and the application rate on the final outcome of the interaction.

Batalla-Carrera et al. [47] evaluated the effect of the co-application of *M. anisopliae* s.s. fungus and *S. carpocapsae*, *S. feltiae*, *Steinernema* sp., and *H. bacteriophora* against the larvae of *Curculio nucum* L.; however, there was neither a synergistic nor antagonistic effect of te pathogen combination on the *C. nucum* larval mortality. The lack of increase in mortality after the joint application was attributed to the fact that fungi alone caused only a low mortality compared with the nematodes alone (22% to 53%), and the effect of fungus in the joint application was diminished due to competition with the nematodes. These results thus suggest that the combination of EPF with EPN is not likely to improve the suppression of *C. nucum* larvae.

Larval instars of the red palm weevil, *Rhynchophorus ferrugineus* Olivier, were targeted by the combination of the fungi *B. bassiana* s.s. and *M. anisopliae* s.s. with the nematode *H. bacteriophora* [48]. In combined treatments, additive and synergistic interactions were recorded, with synergism being more frequent in *H. bacteriophora* combined with *B. bassiana* s.s. in comparison with the combination with *M. anisopliae* s.s. Furthermore, 1–2-week delay in nematode application after the fungus enhanced the efficacy, and generally, combined applications were more effective in early *R. ferrugineus* instars, showing the importance of the stage of the target pest. Overall, the results encouraged the authors to recommend the integration of the delayed application of *H. bacteriophora* after the application of *B. bassiana* s.s. and *M. anisopliae* s.s. in order to achieve successful control of the red palm weevil.

The large pine weevil *Hylobius abietis* L., a major forest pest in northern Europe, was treated through the joint application of the nematodes, *S. carpocapsae* and *Heterorhabditis downesi* Stock, Griffin, and Burnell (Rhabditida: Heterorhabditidae) with entomopathogenic fungi (*B. bassiana* s.s., and *M. anisopliae* s.s.) [49] that were applied on tree stumps to suppress *H. abietis* adult emergence. The effect of the bioagent combination was only additive, with no synergistic action observed. Similarly, no synergy was found in the combination of the fungi *M. brunneum*, *B. bassiana* s.s., and *Beauveria caledonica* Bissett and Widden (Hypocreales: Cordycipitaceae) and nematodes *S. carpocapsae* and *H. downesi* against the same pest, *H. abietis* [50]. While there was a slight additive effect from the co-application, when the agents were applied at half doses, the use of nematodes alone offered good suppression of *H. abietis* populations, and suppression by the mixture did not surpass suppression by EPNs alone. It is likely that the lack of synergy could be because a high mortality caused by the EPNs alone did not leave enough room for improvement by the addition of the fungus. Based on these results, the combination of the tested EPF strains was not recommended, as there would be a need for more effective fungal strains.

### 3.3. Coeloptera Chrysomelidae

To date, the only chrysomelid pest that has been targeted by the combination of the fungi and nematodes is the Colorado potato beetle, *Leptinotarsa decemlineata* (Say). Hussein et al. [18] evaluated the application of *C. fumosorosea* with the nematode *S. feltiae* and recorded an increased efficiency against immature stages of *L. decemlineata* compared with a single biocontrol agent application. The best results were obtained when the nematodes were applied simultaneously with the fungus in comparison with delayed application, which, surprisingly, resulted in an antagonistic effect. Both agents applied alone caused a high pest mortality, and to achieve a synergistic effect, the agents would have to be applied at lower rates. Recently, pupae of the Colorado potato beetle (*L. decemlineata*) were treated with several strains of another fungus, *B. bassiana* s.s., in combination with the nematodes *S. feltiae* and *H. bacteriophora,* also resulting in a higher pupal mortality in comparison with single pathogen application [51]. Similarly to the previous work [18], the mortality achieved with single pathogens was too high to enable the observation of a synergistic effect.

### 3.4. Lepidoptera

Many lepidopteran insects are among the important agricultural pests, and thus a considerable number of studies were performed to evaluate the joint use of entomopathogenic fungi and nematodes. In one of the pioneer studies on the interaction of these pathogens, Barbercheck and Kaya [5] demonstrated that dual infections with the nematode *H. bacteriophora* and fungus *B. bassiana* s.l. had an additive effect, causing a higher level of mortality in the larvae of the beet armyworm, *Spodoptera exigua* (Hübner) (Lepidoptera: Noctuidae). However, no such increase was observed with *S. carpocapsae,* which confirms the effect of pathogen species on the outcome of the interaction.

Shaurub et al. [33] evaluated the effect of the co-application of the nematodes *S. riobrave* and *H. bacteriophora* with the fungus *B. bassiana* s.s., on the last instars of the Egyptian cotton leafworm, *S. littoralis*. All pathogens were applied at the LC25 level, either simultaneously or sequentially, and the synergistic interaction was observed among the different entomopathogen pairings, suggesting that the combination of these entomopathogens could improve the control of *S. littoralis*.

The sugarcane stalk borer, *Diatraea saccharalis* (Fabr.) (Lepidoptera: Crambidae) was exposed to the nematode *H. bacteriophora* nematode in combination with several strains of *M. anisopliae* s.l. of different virulence to the *D. saccharalis* [27]. Interestingly, the positive effect in terms of accelerated pest mortality was present only in the combination of the nematode with the fungal strain of medium virulence. The combination with a highly virulent fungal strain increased the time to death in comparison with the nematodes alone, suggesting an antagonistic effect. It thus demonstrates that the final outcome of the interaction hugely depends on the nature of the fungal strain.

The effect of the dual application of the nematode *H. bacteriophora* with the fungi *M. anisopliae* s.s. and *B. bassiana* s.s. on the larvae of the diamondback moth, *Plutella xyllostella* (L.) (Lepidoptera: Plutellidae), was evaluated in laboratory experiments [52]. The effect of the timing of the nematode application proved to be of an extreme importance as in the hosts treated with both pathogens simultaneously, as well those treated with the nematodes 2 and 4 days after the fungus, the interaction was antagonistic in terms of host mortality, whereas the in the hosts treated with the nematodes 6 days after the fungus, the mortality was higher than the sums of mortalities using the single pathogen applications, and the interaction was thus synergistic. The joint action of same pathogens *H. bacteriophora* and fungi *B. bassiana* s.s. and *M. anisopliae* s.s. against *P. xylostella* was evaluated also both in greenhouse and field conditions [53]. Combined treatments produced the lowest damage percentage on plants and resulted in the highest productivity, with the most efficient combination of *H. bacteriophora* and *M. anisopliae* s.s. The results confirm that the combined use of fungi and entomopathogenic nematodes is an efficient alternative to control diamondback moth.

In a recent study, the nematode *Steinernema yirgalemense* Nguyen et al. (Rhabditida: Steinernematidae) and the fungus *Metarhizium pinghaense* Q.T. Chen and H.L. Guo (Hypocreales: Clavicipitaceae) were found to act synergistically in the control of soil-dwelling life stages of the false codling moth, *Thaumatotibia leucotreta* (Meyrick) (Lepidoptera: Tortricidae), a major pest of citrus plants in South Africa [54]. Interestingly, the interaction between *H. noenieputensis* Malan, Knoetze, and Tiedt (Rhabditida: Heterorhabditidae) and *M. pinghaense* applied simultaneously, and *Steinernema jeffreyense* Malan, Knoetze, and Tiedt (Rhabditida: Steinernematidae) applied 24 h post-fungal application, resulted in antagonistic interactions. This, again, highlights the strongly pathogen species-specific outcome of the interaction. Overall, the study shows the potential of EPN and EPF combination for the control of *T. leucotreta* in citrus orchards in South Africa, which should be further investigated.

### 3.5. Diptera

Four species of EPF, *B. bassiana* s.s., *M. brunneum* s.s., *Cordyceps javanica* (Frieder. and Bally), Samson and Hywel-Jones (Hypocreales: Cordycipitaceae), and *C. fumosorosea* were tested in combination with nematodes *S. carpocapsae* and *S. riobrave* against the pupae of *Rhagoletis pomonella* (Walsh) (Diptera: Tephritidae) [55]. The authors recorded an additive effect on virulence for all fungus–nematode combinations, suggesting that the application of entomopathogenic nematodes and fungi could be an effective option to control *R. pomonella* populations. However, only a combination of *S. riobrave* with *C. javanica* was significantly more virulent than the EPN-only treatments, highlighting the need for proper screening including more EPN and EPF species in order to detect the most efficacious combination. Nevertheless, this study demonstrated, for the first time, that dipteran insect pests can be successfully targeted through the combination of fungi and nematodes.

Another research work evaluated the effects of the combination of fungi and nematodes on fruit flies using *Bactrocera zonata* (Saunders) and *B. dorsalis* (Hendel) (Diptera: Tephritidae) by exposing the larvae, pupae, and pharate adults to *B. bassiana* s.s. and *M. anisopliae* s.s. in combination with *H. bacteriophora* and *S. carpocapsae* in laboratory, glasshouse, and field cage conditions [56]. Interestingly, all combined applications resulted in a greater mortality than individual treatments under all experimental conditions and, in general, *B. bassiana* s.s. was more effective in mixed treatments than *M. anisopliae* s.s. In laboratory tests, many combinations resulted in a synergistic effect, while the interactions in the soil pots, glasshouse, and in the field were mostly additive. Based on the results, similarly to *R. pomonella* [56], fruit flies of the genus *Bactrocera* could be successfully targeted through the combination of entomopathogenic fungi and nematodes.

### 3.6. Other Pest Taxa

In a recent study, the joint application of the nematodes *H. bacteriophora* and *S. feltiae* with the fungi *B. bassiana* s.s. and *M. anisopliae* s.s. resulted in a greater mortality of the onion thrips *Thrips tabaci* Lindeman (Thysanoptera: Thripidae) compared with a single application of each agent, with a prominent additive interaction observed [57]. The field trials confirmed the potential of this combination as the sites with mixed application had lower numbers of thrips larvae and adults, and the damage to the plants was reduced. Therefore, the combined application of entomopathogenic nematodes and fungi could be used for the integrated pest management (IPM) of *T. tabaci*.

### 3.7. Transmission of EPF Spores by Entomopathogenic Nematodes

Various invertebrates can effectively transmit fungal spores in the environment and increase the chances of their encounters with the insect hosts. This phenomenon received attention because of its potential as biocontrol. The first studies focused on the use of pollinating insects [58,59,60], and later the potential of predatory mites was also assessed [61]. Lin et al. [62] developed a method for the control of western flower thrips, *Frankliniella occidentalis* Pergande (Thysanoptera: Thripidae), in which the soil predatory mite *Stratiolaelaps scimitus* Berlese (Mesostigmata: Laelapidae) and the plant-inhabiting mites *Neoseiulus cucumeris* (Oudemans) and *Amblyseius swirskii* Athias-Henriot (Mesostigmata: Phytoseiidae) collected and transported the conidia of the entomopathogenic fungus *B. bassiana* s.s. directly from a commercial rearing substrate.

Recently, Nermuť et al. [63] studied the potential of infective juveniles of entomopathogenic nematodes in the transmission of spores of *C. fumosorosea*. The authors assessed the dispersal of both the conidia and blastospores of *C. fumosorosea* by entomopathogenic nematodes *S. feltiae* and *H. bacteriophora*. The dissemination was studied in agar plates with sand barriers and glass tubes filled with soil in order to simulate the effect of environmental heterogeneity. In agar plates, the dissemination was the highest on clean agar, while the presence of silver sand barriers reduced spore dissemination. Both nematodes dispersed fungal spores through the tubes, with *H. bacteriophora* being more efficient. The authors suggested differences in the presence and persistence of second-stage cuticles or a different foraging behavior of the nematodes could explain this difference. The nematode second stage cuticle was found to be of great importance for spore transmission, as after its removal, the transmission dropped to negligible values. As the authors hypothesized, corrugated and frequently disrupted second stage cuticle probably increases the opportunity for the adhesion of spores that can consequently be transported over a longer distance in the soil environment. In general, blastospores were disseminated more efficiently in comparison with the conidia, which was attributed to the fact that hydrophilic blastospores could better adhere to the nematode cuticle in comparison with the hydrophobic conidia. In general, this study showed a good potential for EPNs to spread EPF spores in the environment.

The study of [64] focused on the role of the nematode foraging strategy on spore dissemination and demonstrated its strong effect because a highly motile “cruiser” strategist *H. bacteriophora* was the most effective at transmission through the soil filled tube, mimicking the conditions deeper in the soil, whereas the soil surface dwelling [65] “ambusher” strategist *S. carpocapsae* was the most effective in the soil column system that was more similar to the soil surface. The experiments with various adhesives showed that sunflower seed oil enhanced spore adhesion to the nematode cuticle and increased spore distribution for all of the tested nematode strains. These findings indicate the potential use of adhesives in pest management to enhance the nematode dissemination of EPF spores, and further research on adhesives optimization, as well as the mechanisms of spore adhesion to the nematode cuticle, may be of importance in the future.

## 4. Conclusions

The combined use of entomopathogenic fungi and nematodes has received increasing attention over the last decades, with many studies appearing from the last few years. Overall, most studies recorded an additive or synergistic effect, while only a small part observed an antagonistic effect (Table 1). Similarly, Koppenhöfer and Grewal [66] stated that nematode–fungus application tends to produce an additive effect. Very often, synergism was not recorded, probably due to the fact that the application rate of the bioagents and, consequently, it’s effect were too high. A synergistic effect is present if the impact of both agents is higher than the sum of the impact from their single applications. For further studies it is recommended that lower doses of bioagents are used. On the other hand, only a few studies have recorded the antagonistic effect of the pathogen combination on the effectiveness of the application (Table 1). It, however, seems that some combinations may have a negative effect on the propagation of both pathogens. Therefore, this aspect should be also taken into account when considering particular nematode–fungus combinations.

The existing research on the joint application of entomopathogenic fungi and nematodes included ca 7 fungal species and 10 nematode species, with most of the studies using the nematodes *H. bacteriophora* and *S. carpocapsae* and the fungi *B. bassiana* s.l. and *M. anisopliae* s.l. (Table 1). Clearly, the studies document t important effect of the bioagent species used on the effectiveness of their combined application. Both in entomopathogenic nematodes and fungi, virulence to a particular host may differ strongly among strains of the same species. For instance, recent research on the pathogens of the Colorado potato beetle *L. decemlineata* revealed that there are both highly virulent and almost nonvirulent strains of the nematode *S. feltiae* [67] and the fungus *B. bassiana* s.s. [68]. Therefore, not surprisingly, a strong effect of the pathogen strain is present in the combination of entomopathogenic fungi and nematodes. Considering such a prominent variability in the performance of various fungal and nematode species combinations, each screening should include the maximum number of pathogen taxa.

It is generally accepted that the timing of the pathogen application had an important effect on the effectiveness of the combined application of the fungi with nematodes, and the effect of the timing can also be pathogen species specific. On the other hand, some studies found no difference between the simultaneous and sequential application of the pathogens. To conclude, the effect of timing is important but very variable, and a general conclusion cannot be made. Therefore, both simultaneous and sequential pathogen application should be compared for each pest–pathogen combination.

To date, most of the research focused on the larval stages of scarabaeids, curculionids, or some lepidopterans. Entomopathogenic nematodes are most often used against soil dwelling insect stages [69], as their infective juveniles can suffer from desiccation and UV radiation [70]. On the other hand, as it has been demonstrated, the combined use of EPNs with EPF was deemed promising for the control of the foliar pests, such as the diamondback moth, *P. xyllostella* [52,53]. Nevertheless, it is likely that soil dwelling insect stages are the primary candidates for nematode fungus applications.

In conclusion, the combined application of nematodes and fungi in biocontrol is a promising strategy that could improve on the environmentally safe control methods of many insect pests. The decisive factor will always be the economic viability, but it could be stimulated by growing public concern about the environmental impact of traditional insecticides, as well as the development of pesticide-resistant pathogens.

## Figures and Tables

**Table 1:** The effects of the joint application of entomopathogenic nematodes with entomopathogenic fungi on agricultural pests.

Target Pest	Nematode *	Fungus *	Interaction	Reference
*Bactrocera dorsalis* (Diptera: Tephritidae)	Hb, Sc	Bb, Ma	Additive, synergistic	[56]
*Bactrocera zonata* (Diptera: Tephritidae)	Hb, Sc	Bb, Ma	Additive, synergistic	[56]
*Coptognathus curtipennis* (Coleoptera: Scarabaeidae)	Hb, Sy	Ma	Additive, synergistic	[45]
*Curculio caryae* (Coleoptera: Curculionidae)	Hi, Sc	Bb, Ma, Cf	Antagonistic, additive	[7]
*Curculio nucum* (Coleoptera: Curculionidae)	Sc, Sf, Hb	Ma	Additive	[47]
*Cyclocephala lurida* (Coleoptera: Scarabaeidae)	Hb, Hm	Bb, Ma	Additive, synergistic	[34]
*Diatraea saccharalis* (Lepidoptera: Pyralidae)	Hb	Ma	Additive, antagonistic	[27]
*Ectinohoplia rufipes* (Coleoptera: Scarabaeidae)	Sc, Hb	Bbr	Additive	[44]
*Exomala orientalis* (Coleoptera: Scarabaeidae)	Sc, Hb	Bbr	Additive	[44]
*Hoplia philanthus* (Coleoptera: Scarabaeidae)	Hb	Ma	Additive, synergistic	[37]
	Hm, Sg	Ma	Additive, synergistic	[6]
*Hylobius abietis* (Coleoptera: Curculionidae)	Sc, Hd	Bb, Ma	Additive	[49]
	Sc, Hd	Mb, Bb, Bc.	Additive	[50]
*Leptinotarsa decemlineata* (Coleoptera: Chrysomelidae)	Sf	Cf	Additive	[18]
	Sf, Hb	Bb	Additive	[51]
*Otiorhynchus sulcatus* (Coleoptera: Curculionidae)	Hb, Sf, Sk	Ma	Additive, synergistic	[8]
	Sk	Ma	Additive, synergistic	[46]
*Plutella xyllostella* (Lepidoptera: Plutellidae)	Hb	Bb, Ma	Antagonistic, additive, synergistic	[52]
	Hb	Bb, Ma	Additive	[53]
*Rhagoletis pomonella* (Diptera: Tephritidae)	Sc, Sr	Bb, Mb, Cj, Cf	Additive	[55]
*Rhynchophorus ferrugineus* (Coleoptera: Curculionidae)	Hb	Bb, Ma	Additive, synergistic	[48]
*Spodoptera exigua* (Lepidoptera: Noctiudae)	Hb, Sc	Bb	Additive	[5]
*Spodoptera littoralis* (Lepidoptera: Noctuidae)	Sr, Hb	Bb	Synergistic	[33]
*Thaumatotibia leucotreta* (Lepidoptera: Tortricidae)	Sy, Sj	Mp	Antagonistic, additive, synergistic	[54]
*Thrips tabaci* (Thysanoptera: Thripidae)	Hb, Sf	Bb, Ma	Additive	[57]

* Nematodes: Hb = *H. bacteriophora*; Hd = *H. downesi*; Hi = *H. indica*; Hm = *H. megidis*; Sc = *S. carpocapsae*; Sf = *S. feltiae*; Sg = *S. glaseri*; Sj = *S. jeffreyense*; Sk = *S. kraussei*; Sy = *S. yirgalemense*. Fungi: Bb = *B. bassiana* s.l.; Bbr = *B. brongniartii*; Bc = *B. caledonica*; Cf = *C. fumosorosea*; Cj = *C. javanica*; Ma = *M. anisopliae* s.l.; Mb = *M. brunneum*.

## Data Availability

The review is based on published data only.
